# Pathogen‐induced maternal effects result in enhanced immune responsiveness across generations

**DOI:** 10.1002/ece3.2887

**Published:** 2017-03-22

**Authors:** Rebeca B. Rosengaus, Nicole Hays, Colette Biro, James Kemos, Muizz Zaman, Joseph Murray, Bruck Gezahegn, Wendy Smith

**Affiliations:** ^1^ Department of Marine and Environmental SciencesNortheastern UniversityBostonMAUSA; ^2^ Department of BiologyNortheastern UniversityBostonMAUSA

**Keywords:** ecological immunology, environmental stressors, hatching success, maternal effects, phenotypic plasticity, transgenerational immune priming

## Abstract

Parental investment theory postulates that adults can accurately perceive cues from their surroundings, anticipate the needs of future offspring based on those cues, and selectively allocate nongenetic resources to their progeny. Such context‐dependent parental contributions can result in phenotypically variable offspring. Consistent with these predictions, we show that bacterially exposed *Manduca sexta* mothers oviposited significantly more variable embryos (as measured by mass, volume, hatching time, and hatching success) relative to naïve and control mothers. By using an in vivo “clearance of infection” assay, we also show that challenged larvae born to heat‐killed‐ or live‐*Serratia*‐injected mothers, supported lower microbial loads and cleared the infection faster than progeny of control mothers. Our data support the notion that mothers can anticipate the future pathogenic risks and immunological needs of their unborn offspring, providing progeny with enhanced immune protection likely through transgenerational immune priming. Although the inclusion of live *Serratia* into oocytes does not appear to be the mechanism by which mothers confer protection to their young, other mechanisms, including epigenetic modifications in the progeny due to maternal pathogenic stress, may be at play. The adaptive nature of maternal effects in the face of pathogenic stress provides insights into parental investment, resource allocation, and life‐history theories and highlights the significant role that pathogen‐induced maternal effects play as generators and modulators of evolutionary change.

## 1 Introduction

Parental investment, resource allocation, and life‐history theories postulate that adults who can accurately perceive their surroundings and anticipate the needs of future offspring based on their own experiences, will be selected to transfer information to their progeny in a context‐dependent fashion (Badyaev, 2008; Mousseau, 1998; Mousseau & Fox, 1998; Sorci & Clobert, 1995; Uller, 2008). Through such tailored contributions, parents can help match the needs of future offspring to the environmental pressures they are likely to encounter (Houri‐Ze'evi et al., 2016; Pigeault, Garnier, Rivero, & Gandon, 2016; Poulin & Thomas, 2008; Sorci & Clobert, 1995; Storm & Lima, 2010). Infectious agents are among the most prevalent environmental cues known to trigger nongenetic maternal and paternal effects (Freitak et al., 2014; Poulin & Thomas, 2008; Sadd, Kleinlogel, Schmid‐Hempel, & Schmid‐Hempel, 2005; Salmela, Amdam, & Freitak, 2015; Trauer‐Kizilelma & Hilker, 2015a, 2015b). Based on their pathogenic history and/or immunological state, parents may protect the next generation by either direct passive transfer of immune function (transgenerational immunity [TGI]) or indirectly through the transfer of molecules that trigger the progeny's own immune system (transgenerational immune priming [TGIP]), rendering offspring less susceptible to the same pathogens experienced by their parents (Freitak et al., 2014; Marshall & Uller, 2007; Pigeault et al., 2016; Roth et al., 2010; Sadd et al., 2005; Sorci & Clobert, 1995). Both TGI and TGIP have been mostly studied at the phenomenological level in a variety of taxonomic groups and appear to be triggered by diverse pathogenic microbes and immune elicitors (Freitak, Heckel, & Vogel, 2009; Grindstaff et al., 2006; Hernández López, Schuehly, Crailsheim, & Riessberger‐Gallé, 2014; Little, O'Connor, Colegrave, Watt, & Read, 2003; Lozano & Ydenberg, 2002; Moret, 2006; Roth et al., 2010; Sadd & Schmid‐Hempel, 2009; Sadd et al., 2005; Tidbury, Pedersen, & Boots, 2011; Trauer‐Kizilelma & Hilker, 2015a, 2015b; Zanchi, Troussard, Martinaud, Moreau, & Moret, 2011; Zanchi, Troussard, Moreau, & Moret, 2012). Although in most of these insect studies the specific molecular mechanisms underlying TGI/TGIP are poorly understood (Freitak et al., 2014; Salmela et al., 2015), recent research has addressed some of the mechanisms underlying heightened immune responsiveness across generations: from the incorporation of bacterial constituents into the developing eggs (Freitak et al., 2014; Knorr, Schmidtberg, Arslan, Bingsohn, & Vilcinskas, 2015; Salmela et al., 2015) to the differential regulation of immune‐responsive genes (Barribeau, Schmid‐Hempel, & Sadd, 2016).

Consistent with the predictions made by parental investment theory, the empirical research on maternal effects, and the facts that immune responses are energetically costly (Bonduriansky, Runagall‐McNaull, & Crean, 2016; Schmid‐Hempel, 2003) and that energy is both limited and limiting, we hypothesized that *Manduca sexta* mothers who experienced pathogenic insults prior to reproduction would make differential contributions toward their future progeny, resulting in phenotypically variable brood (McGinley, Temme, & Geber, 1987; Schmid‐Hempel, 2003; West‐Eberhard, 1989). Furthermore, we also hypothesized that such contributions (including immune‐related products) could be incorporated into eggs resulting in more immune‐competent offspring than progeny of mothers who did not experience such pathogenic pressures. Here, we report on the increased volumetric variability of embryos when mothers were exposed to pathogenic bacteria during or soon after oogenesis. Moreover, maternal treatment impacted both embryonic developmental timing and hatching success as well as immune responsiveness of first instar larvae born to mothers treated with heat‐killed or live bacteria, relative to larvae from control mothers.

## 2 Materials and Methods

### 2.1 Bacterial and insect cultures, and pupal injections

**Figure 1:**
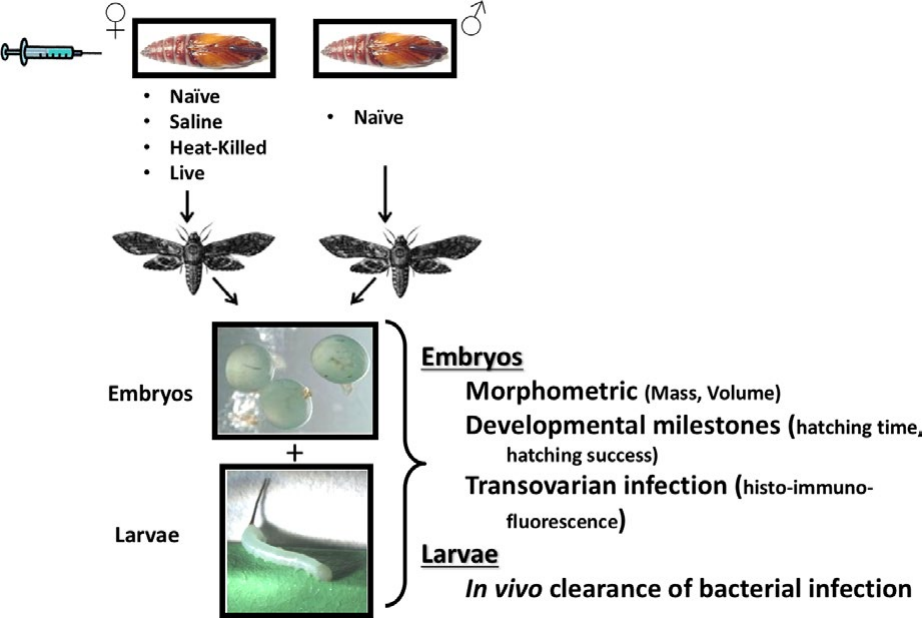
Schematic of research protocol

The Gram‐negative bacterium, *Serratia marcescens*, was chosen to elicit a maternal immune response because it is an ecologically relevant pathogen, commonly found on foliage and in soil (Sikorowski, Lawrence, & Inglis, 2001). For further details on the choice of pathogen and the rationale behind our experimental design, see Appendix S1. *Manduca sexta* fertilized eggs (i.e., embryos) and larvae were originally obtained from Carolina Biological Supply (Burlington, NC). Larvae were reared on standard artificial diet (Bell & Joachim, 1976) at 25°C under a 16‐hr:8‐hr light/dark cycle and were injected as pupae 2 days prior to their expected date of eclosion. A subset of female pupae were randomly weighed, and all female pupae were allocated to one of the following four different treatments (Figure 1): naïve (unmanipulated), saline injection (injected with 10 μl of sterile Burns‐Tracey saline [BTS]), heat‐killed bacterial injection (injected with 10 μl of a 10^8^/ml heat‐killed *S. marcescens* in BTS), and live‐bacteria injection (injected with a nonlethal total dose of 4,000 live *S. marcescens* bacterial cells suspended in 10 μl of BTS). Although heat‐killed‐*Serratia*‐treated mothers received a larger dose of immune elicitors relative to the live‐*Serratia*‐injected mothers, such dose increased the probability that the elicited physiological responses in the mothers had “trickle‐down” consequential effects across generations without the added negative impact of disease itself. The maternal naïve treatment served as an important second control to identify the effects of maternal stress associated with cuticular puncturing. Male pupae always remained untreated (naïve). Before injection, all females’ abdominal side was swabbed with 70% ethanol and injections were performed by inserting the sterile needle of a 10‐μl Hamilton syringe between the fourth and fifth ventral abdominal segments of the pupa. Following injection, the area was swabbed with 70% ethanol for a second time, and immediately after, male and female pupae were placed at the base of flying cages. Although a slight possibility existed that alcohol swabbing did not completely sterilize the cuticle before injections, great care was given to ensure that no contaminant bacteria other than our *Serratia* strain was injected into the pupa. For studies in which identification of both parents was required (morphometric experiments [mass and volume data], see below), individual treated females were mated with individual untreated males in cages (30 × 30 × 60 cm). For all additional experiments, groups of 4–6 identically treated females were mated with similar numbers of untreated males in larger breeding cages (40 × 40 × 75 cm). Although the parentage in these cages could not be determined, such breeding groups resulted in a more efficient production of known same‐aged embryos. Upon eclosion, parents were provided with 10% sucrose as a food source. The mated moths oviposited on 30 mm foam plugs infused with tobacco extract and suspended from the cage ceiling. Embryos were then collected and allocated to the different experiments below.

### 2.2 Morphometric analyses of embryos

Embryonic mass and volume measurements were used as proxy for embryo quality (Rossiter, 1991). While embryonic mass data were collected across a total of 61 mothers (naïve = 15, saline = 18, heat‐killed *Serratia* = 16, live *Serratia* = 12), embryonic volume was collected from a total of 59 mothers, mostly the same mothers from which we collected embryonic mass (naïve = 13, saline = 16, heat‐killed *Serratia* = 18, live *Serratia* = 12). Mothers usually oviposit for up to 3 days posteclosion, and all of their embryos were collected throughout this time period. Thus, our protocol captured the entire reproductive output of each female/maternal treatment. Each embryo was individually weighed and photographed under a 4× magnification (SPOT Q camera). Given that embryos are spherical, we used the cross‐sectional area of the embryo image to calculate its volume by using the formula of a sphere *V* = 4/3(π*r* ^3^). Out of a total of 3,720 collected embryos, we had both mass and volume data for 2,250 embryos across all four maternal treatments (naïve = 499, saline = 540, heat‐killed‐*Serratia* = 377 and live‐*Serratia* injected = 834).

### 2.3 *In vivo* bacterial clearance assay: microinjections, bleeding, and enumeration of recovered bacteria

A clearance of infection assay was developed in which we not only determined the ability of larvae to reduce/eliminate (i.e., clear) a *Serratia* infection *in vivo*, but also established the time course for eradicating of such infection. Embryos from saline (*n = *90), heat‐killed (*n *=* *63) and live‐*Serratia* (*n *=* *75) maternal treatments were placed on insect diet shortly after oviposition. These embryos and their mothers were a different subset than those used for the morphometric analyses described above. Because the aim of the study was to test whether maternal exposure to heat‐killed or live bacteria resulted in more immune competent progeny relative to the offspring of saline‐injected mothers, this experiment did not include larvae of naïve mothers. Two days posthatching, the larvae were cold‐immobilized, swabbed with 70% ethanol, and then injected with a live *Serratia* (a total of 228 larvae across the three treatments) through the ventral intersegmental membranes with a pulled glass capillary tube (Fig. S1). The challenge dose/larva was 1.5 × 10^5^ live *Serratia* cells suspended in 1 μl of BTS, administered using a microinjection apparatus (Picospritzer). Additionally, to test whether our *Manduca* laboratory colony was naturally infected with *Serratia*, first instar larvae from naïve mothers were also injected with 1 μl of sterile BTS lacking *Serratia* (*n *=* *40). To visually confirm that both the bacterial and control saline solutions successfully reached the hemocoel, both solutions were colored with sterile red food coloring (1 μl of dye/40 μl of BTS). The food coloring was metabolized within the first 24 hr with no apparent negative effects (Rosengaus, Malak, & McKintosh, 2013). Subsequently, larvae were placed in labeled cups containing nutrient medium. A subset of these insects were bled either immediately (providing a baseline *Serratia* recovery rate) or at 2, 8, 12, 24, 36, and 48 hr postchallenge to enumerate the number of *Serratia* cells as time postchallenge progressed (Figs S2 and S3). Hemocoel washes (40 μl/larva/plate, two plates per larva) containing recovered *Serratia* cells were seeded over the surface of solidified tryptic soy agar plates. These samples were spread over the entire plate using sterile glass beads. The plates were incubated at 25°C for at least 24 hr (Figure 3 for additional detailed protocols). At this temperature, *Serratia* typically grows as a pink/red bacteria due to the expression of the prodigiosin gene (de Araújo, Fukushima, & Takaki, 2010; Khanafari, Assadi, & Fakhr, 2006). All pink/red bacterial CFUs were counted (see additional details in the Appendix S1). These bleeding and plating techniques recovered both non‐*Serratia* bacteria (mostly white smooth CFUs) naturally colonizing the insect's hemocoel and *Serratia* (Fig. S4). Because the red pigmentation of CFUs was the sole identifier for the enumeration of recovered *Serratia*, it was imperative we ensured that white CFUs were not mutant *Serratia* strains (due to possible mutations in the prodigiosin gene, hence white). To this end, we used PCR to confirm that the white bacteria growing on our plates were not *Serratia* by using *Serratia*‐specific primers (LuxS: forward primer: 5′‐TGCCTGGAAAGCGGCGATGG‐3′; reverse primer: 5′‐CGCCAGCTCGTCGTTGTGGT‐3′ primers; Joyner, Wanless, Sinigalliano, & Lipp, 2014; Fig. S5). These PCR experiments gave us confidence that the enumeration of only red/pink CFUs provided an unbiased count of recovered *Serratia*.

### 2.4 Developmental milestones and hatching success of progeny as a function of maternal treatment

To determine the impact that maternal treatment had on developmental milestones of offspring (e.g., hatching time and their hatching success), we followed the development of a total of an additional set of 3,275 embryos (*n *=* *834 embryos from naïve mothers, 604 from saline‐injected mothers, 973 embryos from heat‐killed‐bacteria‐injected mothers and 864 from live‐bacteria‐injected mothers). This set of embryos was different from those used in the morphometric and clearance of bacteria assays. Within the first 24 hr after the onset of oviposition, each embryo was individually coded and maintained on *Manduca* diet in 96‐well cell culture plates. These embryos were checked daily for a maximum of 7 days, and the number of days elapsed from oviposition to hatching and their hatching success were quantified.

### 2.5 Maternal translocation of bacteria during oogenesis

We investigated whether live *Serratia* (and/or its constituents) were incorporated into the embryo via transovarian transmission, a phenomenon reported in both *Galleria mellonella* and *Tribolium castaneum* (Freitak et al., 2014; Knorr et al., 2015). To this end, embryos (*n *=* *18 from up to seven different live‐bacteria‐injected mothers) were frozen, and 20 μm cryosections were cut and collected onto positively charged Thermo Scientific™ Shandon™ ColorFrost™ Plus Slides. The resulting 16–20 sections/embryo permitted observation of the entire embryo. Further description of this protocol along with the preparation of positive controls is available in Appendix S1.

### 2.6 Statistical analyses

Given that the main aim of our work was to test whether immune elicitation (by components of the heat‐killed *Serratia* or live bacteria) affected progeny's phenotype, analyses focused on how measures of embryo quality and immune responsiveness varied between each of the maternal treatments (naïve, heat‐killed, and live *Serratia*) and the reference saline‐injected maternal treatment. Neither embryo mass (all Shapiro–Wilk statistics >.92 and their corresponding significance *p *<* *.0001 across the four maternal treatments), embryo volume (all Shapiro–Wilk Statistic >.87 all *p *<* *.0001), nor numbers of recovered bacteria (all Shapiro–Wilk Statistics >.55, all *p *<* *.0001) were normally distributed despite attempts to normalize the data through log_10_, log_*n*_, and arcsin transformations. Hence, data were analyzed using several nonparametric tests.

Embryonic mass and volume data across the four maternal treatments were subjected to a Levene test for homogeneity of variance. Overall differences in the median embryonic mass and volume were also analyzed using Kruskal–Wallis (KW) tests across the four treatments with subsequent Mann–Whitney (MW) tests between treatments and a Bonferroni correction due to multiple pairwise comparisons (setting a conservative significance threshold of *p *≤* *.01). Unfortunately, this latter analysis could not control for the effect of maternal identity nor for the fact that multiple embryos originated from the same mother. Hence, we generated two different linear mixed‐effect models based on 2,250 embryos for which we had both mass and volume data. The first model tested the effect of maternal treatment (fixed categorical variable) on embryo mass while including maternal identity as a random‐effect term and embryo volume as a fixed covariate. The second model similarly tested the effect of maternal treatment on embryo volume while controlling for embryo mass (fixed covariate) and while accounting for the nonindependence of the observations and residuals obtained from the same mothers (random effect).

Both models focused on main effects and interactions between the maternal treatment and embryo volume or maternal treatment and embryo mass, respectively. Pairwise comparisons between maternal treatments were also generated as part of these linear mixed‐effect models while using a Bonferroni correction.

Differences in the rates of *Serratia* recovery as a function of maternal treatment (while controlling for time of bleeding) were also analyzed with a GLM that included maternal treatment as a categorical factor and time of bleeding as a scalar covariate. Pairwise differences in the mean rank (MR) recovery rates of *Serratia* between incubation time points postchallenge as a function of maternal treatment were compared with MW tests (after Bonferroni corrections set a threshold value of *p *≤* *.002).

Differences in hatching success as a function of maternal treatment were analyzed using three different statistical tests: (1) A Kaplan–Meier test generating the median number of days elapsed from oviposition to hatching and the Breslow statistic (with the significance level adjusted via Bonferroni corrections due to multiple pairwise comparisons) which compared the overall differences in the probability and time course of hatching; (2) the Cox proportional regression model which allowed testing for differences in hatching time while controlling for the effects of the two scalar covariates embryonic mass, volume, and the categorical factor maternal treatment; and finally; (3) hatching success on day seven postoviposition was analyzed by using a 4 × 2 χ^2^ test. All analyses were carried out using SPSS version 22.

## 3 Results

### 3.1 Morphometric analyses of embryos

**Figure 2:**
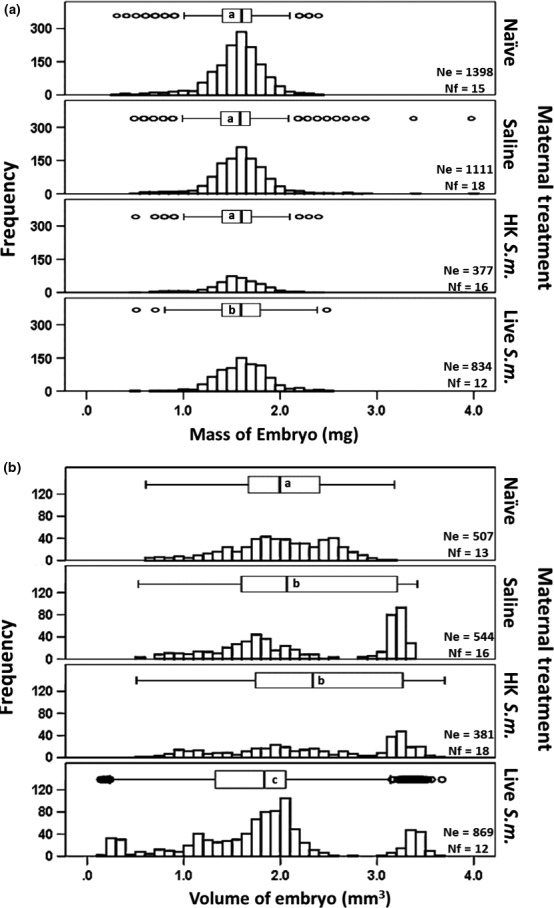
Frequencies of distribution for embryo mass (a) and embryo volume (b) as a function of maternal treatment. Ne indicates the number of embryos and Nf the total number of mothers treated. Different letters within the boxplots above the frequencies of distributions represent significant differences in pairwise comparisons between each of the three maternal treatments and the saline controls (after a Bonferroni correction at *p *≤* *.01). While overall differences in embryo mass were significant (χ^2^ = 18, *df* = 3, *p *<* *.0001, Kruskal–Wallis [KW]), pairwise comparisons (by Mann–Whitney [MW] test) between the naïve and saline treatments (*U* = 752,323, *Z* = −1.3, *p *>* *.05) as well as the heat‐killed and saline treatments (*U* = 198,398, *Z* = −1.5, *p *>* *.05) were not significantly different. Only the mass of embryos from live‐*Serratia*‐injected mothers was significantly different from that of the saline maternal treatment (*U* = 433,925, *Z* = −2.4, *p *=* *.01). Volume of embryos was significantly different across all four maternal treatments (χ^2^ = 105, *df* = 3, *p *=* *.0001, KW). Only the pairwise comparisons (by MW test) between naïve versus saline‐injected mothers (*U* = 115,385, *Z* = −4.6, *p *=* *.0001) and live‐*Serratia‐* versus saline‐injected mothers (*U* = 189,599, *Z* = −6.3, *p *=* *.0001) were significant. Differences in embryonic volume between heat‐killed‐bacteria‐ and saline‐injected mothers were not (*U* = 96,884, *Z* = −1.7, *p *=* *.09)

The variance in embryonic mass (Levene statistic = 9.7, *df*_1_ = 3, *df*_2_ = 2,246, *p *<* *.0001; Figure 2a) and embryonic volume (Levene statistic = 54.4, *df*_1_ = 3, *df*_2_ = 2,246, *p *<* *.0001; Figure 2b) across the four maternal treatments was not homogeneous. Yet, embryonic mass data clustered more tightly around the median than embryonic volume, which exhibited broader dispersion (Figure 2a,b). Relative to the volume of embryos from naïve (from 0.6432 to 3.1722 mm^3^, range = 2.53, MR = 1,138.1) and saline‐injected mothers (from 0.5856 to 3.3971 mm^3^, range = 2.81, MR = 1,266.0), the embryos from heat‐killed‐*Serratia*‐injected mothers had a wider range of volumes (from 0.5589 to 3.6086 mm^3^, range = 3.05, MR = 1,365.0). This measure of offspring phenotypic variability was particularly pronounced in the embryos of live‐*Serratia*‐injected mothers (from 0.1930 to 3.6987 mm^3^, range = 3.50, MR = 992.7) which exhibited significantly lower medians than embryos from saline‐injected mothers (see Figure 2b for statistical details). Interestingly, the mere act of aseptically puncturing the maternal pupal cuticle can elicit cross‐generational effects on embryo volume (Figure 2b) but not embryo mass (Figure 2a): Differences in the median embryonic volume between progeny of naïve (MR = 481.6) and saline‐injected (MR = 567.4) mothers were significantly different (*U* = 115,385, *Z* = −4.6, *p *<* *.0001, MW, Figure 2b). Further detailed analyses showed that embryos larger than 3 mm^3^ (Figure 2b) were not all progeny of a single large mother. These more voluminous embryos were progeny of between 12% to 16% of the females across the various maternal treatments. These same mothers (not necessarily the heaviest) also produced a range of smaller embryos, including embryos that were as small as 1.0–1.6 mm^3^ (e.g., in the heat‐killed *Serratia* treatment).

The use of mixed‐effect models allowed accounting for the nonindependence of the observations and residuals obtained from the same mothers. The first model showed that all variables significantly influenced embryo mass (overall *F* = 18.0, *df*_numerator_ = 3, *df*_denominator_ = 2,242, *p *≤* *.0001, *n* = 2,250 embryos): Maternal treatment was a significant predictor (*F* = 1,555.2, *df*_numerator_ = 3, *df*_denominator_ = 2,242, *p *≤* *.0001) of embryonic mass even after controlling for the effect of embryo volume (*F* = 271, *df*_numerator_ = 1, *df*_denominator_ = 2,242, *p *≤* *.000) and maternal identity (random effect). Relative to the mass of embryos of saline‐injected mothers (mean mass in mg [±SE] = 1.591 [±0.013]), embryos of naïve (mean mass [±SE] = 1.526 [±0.013]), heat‐killed *Serratia* (mean mass [±SE] = 1.524 [±0.016]), and live *Serratia* (mean mass 1.631 [±0.01] mg) were all significantly different (all at *p *≤* *.02 after a Bonferroni correction, see Table S1 for these and additional pairwise comparisons between all maternal treatments, Appendix S1). There was a significant difference in the interaction between maternal treatment and embryo volume (*F* = 31.6, *df*_numerator_ = 3, *df*_denominator_ = 2,242, *p *≤* *.0001).

With respect to embryo volume, a second linear mixed‐effect model showed that both maternal treatment and embryo mass significantly influenced embryo volume (overall *F* = 65.8, *df*_numerator_ = 3, *df*_denominator_ = 2,242, *p *≤* *.0001, *n* = 2,250 embryos). Maternal treatment was a significant predictor of embryonic volume (*F* = 41.3, *df*_numerator_ = 4, *df*_denominator_ = 2,242, *p *≤* *.05), even after controlling for the effect of embryo mass (*F* = 175.5, *df*_numerator_ = 1, *df*_denominator_ = 2,242, *p *≤* *.0001) and maternal identity (random effect). Relative to the volume of embryos of saline‐injected mothers (mean volume in mm^3^ [±SE] = 2.270 [±0.032]), embryos of naïve (mean volume [±SE] = 2.054 [±0.034]) and live‐*Serratia*‐injected mothers (mean volume = 1.819 [±0.026]) were significantly different (at *p *≤* *.0001). The volume of embryos from heat‐killed‐*Serratia‐*injected mothers (mean volume [±SE] = 2.379 mm^3^ [±0.038]) did not differ significantly relative to their counterparts from the saline maternal treatment (mean volume in mm^3^ [±SE] = 2.270 [±0.032], *p *=* *.2]). Additional pairwise comparisons (with Bonferroni corrections) between all treatments generated through the linear mixed‐effect model are given in Table S2. There was no significant interaction between maternal treatment and embryo mass (*F* = 1.5, *df*_numerator_ = 3, *df*_denominator_ = 2,242, *p *≤* *.2).

### 3.2 Developmental milestones and hatching success of progeny as a function of maternal treatment

**Figure 3:**
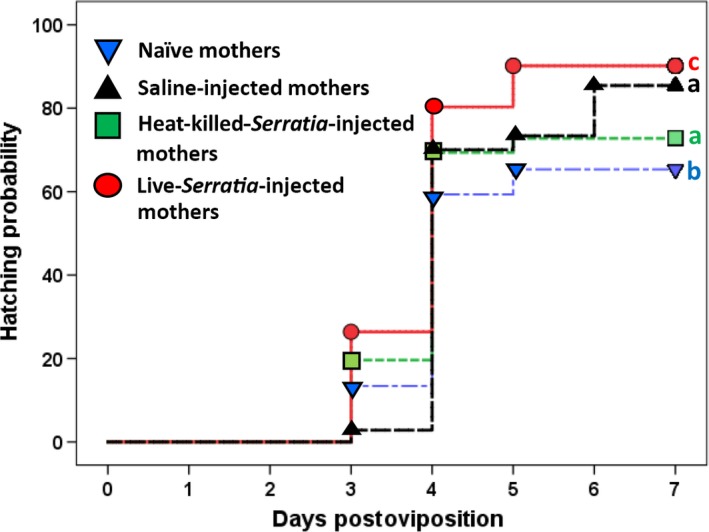
Hatching time course of embryos from different maternal treatments. Different letters to the right of each line represent significant differences in pairwise comparisons between the time course of hatching (by Breslow statistic following a Bonferroni correction). By the seventh day postoviposition, hatching success (measured as percent number of embryos hatched) was significantly different across the maternal treatments (4 × 2 χ^2^, see text)

After controlling for the effects of embryonic mass and volume (maternal identity and mass were not available), the time course of hatching was significantly impacted by maternal treatment (Wald χ^2^ = 77.8, *df* = 3, *p *<* *.0001, Cox proportional regression), with embryos from live‐*Serratia*‐injected mothers having consistently higher hatching success than offspring from any of the other maternal treatments (Figure 3). Although the median hatching time of four days across all treatments was not significantly different (Kaplan–Meier test), the live‐bacteria maternal treatment had more than ~20% of embryos hatching a full day earlier (day 3 postoviposition) than the majority of embryos from the other treatments (day 4 postoviposition, Figure 3). By the end of the seven‐day census period, percent hatchability was significantly different across the four maternal treatments (χ^2^ = 153.8, *df* = 3, *p *<* *.0001; 4 × 2 χ^2^ test): The progeny of live‐bacteria‐injected mothers had the highest hatchability (up to 90% relative to the naïve, saline, and heat‐killed treatments = 68.3%, 85.4%, and 72.9%, respectively; Figure 3). The higher hatching success of embryos of saline‐ and heat‐killed‐*Serratia*‐injected mothers together with their increased volume variability relative to the naïve mothers (Figure 2b) strongly support the view that maternal stress (resulting from cuticular injury) also fosters fitness‐related effects across generations. The fact that hatching success was highest for embryos of live‐*Serratia*‐treated mothers indicates that effects on progeny developmental time, when their mothers face live pathogens, exceed those caused by cuticular injury.

### 3.3 Clearance of infection assay

Accurate enumeration of recovered bacteria from the larvae's hemocoel required first a positive identification of *Serratia*. The use of *Serratia‐*specific primers corroborated that counting only the pink/red bacteria would represent reliable recovery rates of *Serratia* used in the larval challenges. None of the DNA from white CFUs growing in our plates was amplified by the *Serratia*‐specific primer (Fig. S4). Positive controls for the PCR assays included one pink CFU grown from our experimental frozen bacterial stock. We also confirmed that our laboratory *Manduca* colony was not naturally infected with this microbe as hemolymph of saline‐injected larvae (who were progeny of naïve mothers) never showed positive growth for *Serratia*.

**Figure 4:**
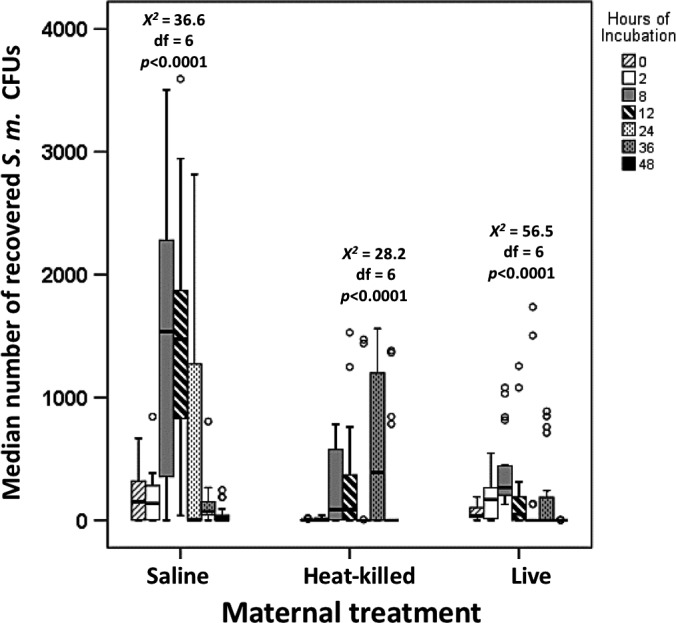
Median number of recovered *Serratia* CFUs (±interquartile) as a function of both maternal treatment and time elapsed as larvae underwent a challenge with live bacteria. The circles indicate outliers. Statistical analyses comparing overall *Serratia* loads at different time periods within each maternal treatment were performed with Kruskal–Wallis tests. Table S3 indicates significant differences in all possible pairwise comparisons between two incubation time periods (by Mann–Whitney *U* test) after Bonferroni corrections within each of the maternal treatments

Results from our GLM test helped assess the impact that maternal treatment had on the larvae's ability to clear bacterial infection. We analyzed two complementary measures of an insect's immune responsiveness: the number of *Serratia* cells circulating in the larva's hemocoel (bacterial loads) and the timing of eradication of bacteremia. Maternal treatment was a significant and independent predictor of the number of recovered *Serratia* (Wald χ^2^ = 61.0, *df* = 2, *p *<* *.0001, Figure 4). Bleeding time also significantly affected the number of recovered *Serratia* from the insect's hemocoel (Wald χ^2^ = 21.5, *df* = 1, *p *<* *.0001, Figure 4). This variable also interacted with maternal treatment (Wald χ^2^ = 22.2, *df* = 2, *p *<* *.0001). The recovery rate of *Serratia* cells from progeny of saline‐injected mothers represented the expected bacterial growth dynamics within the insect host. The injected bacteria appeared to have multiplied within the larvae reaching a maximum load at approximately 8 and 12 hr postchallenge (Fig. S5). By 36 and 48 hr, the microbial loads had been reduced considerably, although some *Serratia* were still recovered (Fig. S5). The progeny of heat‐killed (MR = 31.2)‐ and live‐*Serratia* (MR = 32.5)‐injected mothers had significantly lower bacteria loads at 24 hr postchallenge relative to challenged larvae who were progeny of saline‐injected mothers (MR = 46.7, overall across the three maternal treatments χ^2^ = 11.8, *df* = 2, *p *=* *.003, KW). Surprisingly, larvae from the heat‐killed maternal treatment exhibited an abrupt significant surge of recovered *Serratia* from the 24 (MR = 15.8, *n* = 20) to 36 hr (MR = 23.6, *n* = 18) postchallenge (*U* = 107.0, *Z* = −2.5, *p *=* *.01, MW) followed by a borderline significant reduction in bacteria between the 36‐ to 48‐hr period (MRs = 21.4 [*n* = 18], and 15.6 [*n* = 18], respectively, *U* = 110, *Z* = −1.8, *p *=* *.06, MW, Figure 4). Within the live‐*Serratia* maternal treatment, differences in the recovery rates of *Serratia* between 24 hr (MR = 21.5, *n* = 22) and 36 hr (MR = 23.5, *n* = 22) were not significantly different (*U* = 220, *Z* = −0.7, *p *=* *.5, MW), nor were the differences between the 36 (MR = 20.6, *n* = 22) and 48 hr (MR = 18.0, *n* = 16) postchallenge (*U* = 152, *Z* = −0.9, *p *=* *.5, MW, Figure 4; see Table S3 for additional pairwise comparisons).

### 3.4 Maternal translocation of *Serratia*

Of the total 18 immunofluorescently stained samples, none showed positive fluorescence for *S. marcescens* in any region of the embryo (Fig. S6). We are confident that our protocol would have detected high concentrations of *S. marcescens* based on the strong signal seen in our positive controls. However, we realize that minute amounts of *S. marcescens* may have escaped detection.

## 4 Discussion

*Manduca sexta* has been a long‐standing test organism for the study of insect immunity (Kanost & Blissard, 2015; Kanost, Jiang, & Yu, 2004; Zhen, Najar, Wang, Roe, & Jiang, 2008 and references therein). We feel confident that both cuticular injury and, particularly, the exposure to immune elicitors or live *Serratia* set the stage for physiological trade‐offs between a mother's somatic/immune maintenance and her reproductive system (see Appendix S1 for analyses on impacts of maternal treatment on maternal biological measures). Using this tractable model species, we focused on whether pathogen‐induced maternal effects resulted in increased phenotypic variability of their progeny in terms of embryological mass, volume, developmental milestones as well as immune competency in first instar larvae.

### 4.1 Morphometric analyses of embryos

Although overall, embryonic mass and volume were both influenced by maternal treatment, embryo mass appears to be a more constrained physical attribute than embryo volume (Figure 2a,b). The fact that these two morphometric measures of embryo quality respond differently to maternal pathogenic stress suggests that, just like in the gypsy moth (Rossiter, 1991), mass of *Manduca* embryos may not be as plastic and, hence, may not be a reliable measure of differences in maternal provisioning, embryonic metabolic state (Maino, Kearney, Nisbet, & Kooijman, 2014), developmental transformation (Maino & Kearney, 2014), embryo quality, and, ultimately, fitness. Interestingly, the mass and volume of embryos oviposited by saline‐injected mothers (even after controlling for maternal identity and after accounting for the fact that multiple embryos originated from the same mother) were significantly different from embryos of naïve mothers (Figure 2b, Tables S1 and S2 pairwise comparisons). This suggests that the mere act of aseptically puncturing the cuticle of the maternal pupal case elicits cross‐generational phenotypic effects. Cuticle abrasion and wounding are known to elicit immune responses (Brey et al., 1993; Johnston & Rolff, 2013) which are mediated by the JAK/STAT and JNK pathways (Razzell, Wood, & Martin, 2011). Thus, recognizing that maternal effects are inducible by cuticular injury is important if we are to separate the effects of immune elicitation (via lipopolysaccharides, peptidoglycans, and/or actively dividing live bacteria) from cuticular wounding. The progressively increased range of embryo volumes from the naïve to live‐injected mothers (Figure 2b) indicates that *Serratia* immune elicitation in heat‐killed‐ and live‐bacteria‐injected mothers, foster additional variability that surpasses that caused by cuticular wounding. The production of less voluminous embryos is likely the result of trade‐offs between competing maternal needs (Moreau, Martinaud, Troussard, Zanchi, & Moret, 2012): maternal immune responsiveness versus reproduction. Given that energy is limiting, mothers who divert energy away from oogenesis to instead generate their own immune responses could conceivably produce less voluminous embryos. Yet, trade‐offs would not explain why some heat‐killed‐ and live‐bacteria‐injected mothers also produced some of the most voluminous embryos (Figure 2b). The bias toward more voluminous embryos in both the heat‐killed‐ and live‐*Serratia*‐treated mothers may have resulted from a “bet‐hedging” strategy (i.e., parental investment decisions) whereby immune‐elicited mothers made disproportionately larger contributions (i.e., larger investment) to some embryos at the expense of others (Rossiter, 1991). Our results are consistent with the notion that immune elicitation and, in particular, the presence of live bacteria in mothers affect embryo mass and particularly embryo volume above and beyond the maternal effects induced by cuticular wounding.

### 4.2 Developmental milestones as a function of maternal treatment

Maternal treatment not only influenced physical attributes of the embryos but also impacted their developmental timing and hatching success. A higher proportion of progeny from live‐bacteria‐injected mothers hatched on the third day postoviposition relative to progeny from the other treatments. This consistent pattern across the 7‐day census period translated into a 90% hatchability rate. Just as in the morphometric experiment, our hatchability experiment shows that exposure to live bacteria during oogenesis appears to hasten the development of embryos beyond the effects recorded for heat‐killed‐bacteria‐ and saline‐injected mothers. The fact that progeny of the latter two maternal treatments had faster development and higher hatchability than embryos from naïve mothers points to differential maternal contributions when facing general stress (e.g., cuticular wounding). Hence, pathogenic stress (in the form of sublethal dosages of live bacteria) appears to foster further maternal investment. Our results point to the following interesting questions: What are the putative maternal contributions that speed developmental time and increase embryo hatchability when the maternal immune system is activated during oogenesis? And what compounds could mothers contribute that result in larvae clearing bacterial infection faster? Do the contributed compounds in the face of pathogenic stress have pleiotropic effects and thus impact simultaneously embryo development, hatching, and immune responsiveness of progeny? Or are each of these different compounds tailored to influence separately developmental timing and immune function? Possibly, mothers facing pathogenic stress provision their progeny with more of the important metabolites [e.g., carbohydrates, protein and lipids (Kinsella, 1966; Pant, Kumar, & Dhar Singh, 1979)] which could result in faster development and higher hatchability. All the above questions remain to be answered.

### 4.3 Clearance of infection assay

In addition to the pathogen‐induced volumetric maternal effects as well as the effects influencing hatching time and hatching success, our results also indicate that mothers who face immune elicitation can alter the immune competency of their progeny: Larvae of heat‐killed‐ and live‐*Serratia‐*injected mothers supported significantly lower *Serratia* loads and cleared the infection earlier than progeny of saline‐injected mothers. The surge of recovered *Serratia* during the 24 to 36 hr postinjection in progeny from heat‐killed‐ and live‐bacteria‐injected mothers (Figures 4 and S5) points to the possibility that bacteria at 36 hr postinjection were descendants of persister cells (Lewis, 2007) which were dormant at the 24‐hr hemolymph collection time. Interestingly, some bacteria are known to evade host's immune defenses by “hiding” within the same immune‐related phagocytic cells (i.e., hemocytes) whose sole function is the destruction of said pathogenic cells (McGonigle, Purves, & Rolf, 2016). Alternatively, the surge in *Serratia* loads at 36 hr may have corresponded to the time when the larval hemocytes and/or antimicrobial peptides had been depleted due to the successful control of bacteremia at the 24 hr postchallenge mark.

There are several putative mechanisms, not necessarily mutually exclusive, that can explain the apparent heightened immune competency of progeny from heat‐killed‐ and live‐*Serratia*‐injected mothers. First, the translocation of bacteria and/or bacterial components from *Serratia*‐treated mothers to oocytes could elicit the embryo's own immunological responses in a strain‐specific manner (Freitak et al., 2014; Knorr et al., 2015). We found no evidence for this mechanism (Appendix S1). Second, mothers could incorporate prefabricated nutrients and immune‐related compounds into the developing oocytes (i.e., antimicrobial peptides, mRNAs, RNA‐binding proteins, enzymes), a strategy that could presumably protect progeny against microbial challenges until the offspring's own transcriptional machinery becomes active (Sysoev et al., 2016). These maternal “gifts” have been reported in both invertebrate and vertebrate species (Broggi, Soriguer, & Figuerola, 2016; Grindstaff, Brodie, & Ketterson, 2003; Hasselquist & Nilsson, 2009; Moret, 2006; Rossiter, 1991; Sadd & Schmid‐Hempel, 2007; Seppola, Johnsen, Mennen, Myrnes, & Tveiten, 2009; Trauer‐Kizilelma & Hilker, 2015a, 2015b; Zanchi et al., 2012). Finally, mothers could modulate the progeny's gene expression through DNA methylation and/or histone acetylation (Oldroyd et al., 2014) which could result in the lower microbial loads and earlier eradication of *Serratia* in offspring of heat‐killed and live‐injected *Serratia*. Notably, some of the above mechanisms proposed to explain maternal effects in the face of disease can also apply to instances where paternal effects occur (Rodgers, Morgan, Leu, & Bale, 2015). Fathers could transmit directly and/or indirectly (through the female) information to their unborn progeny in the form of epigenetic changes or immune‐related compounds via their seminal fluids. Establishing whether maternal and paternal effects are additive or multiplicative may help identify environmental reasons underpinning phenotypic differences (i.e., plasticity) and their evolutionary consequences (Senner, Conklin, & Piersma, 2015; West‐Eberhard, 1989).

Our findings demonstrate the significant role that pathogens play as both agents of selection and generators of phenotypic variability. The recognition that environmental pressures (including pathogenic burden) can impact life‐history traits across generations has represented a major paradigm shift in our understanding of the interdependence among environmental stress, physiological responses, inheritance as well as rates of evolutionary innovation and change (Houri‐Ze'evi et al., 2016; West‐Eberhard, 1989). Hence, this is a fertile area that merits further research.

## Conflict of Interest

We declare we have no competing interests.

## Authors’ Contributions

RBR and WS conceived, designed, ran statistical analyses and coordinated the study. They also drafted the manuscript. NH performed both the clearance of bacteria assay and the histoimmunofluorescence experiments and with the help of CB, enumerated and verified the identity of the enumerated bacteria. JK, JM, and MZ raised, collected, and quantified *M. sexta* embryos used in this study and assisted in data analyses. JM and BG performed developmental and hatching success studies. All authors gave final approval for publication.

## Ethics

*Manduca sexta* were purchased from Carolina Biological Supply, and these research organisms do not require animal ethics approval. To avoid pain, animals were cold‐immobilized before any injections were performed.

## Data Accessibility

Data presented in this paper are available as an electronic Appendix S1.
