# Supplementary material for: Pathogen‐induced maternal effects result in enhanced immune responsiveness across generations
Source: Ecol Evol. 2017 Mar 22;7(9):2925–35. doi: 10.1002/ece3.2887 (PMC5415515; doi:10.1002/ece3.2887)
Supplement: Supplementary file 1 [file ECE3-7-2925-s001.docx]

Supporting information

Pathogen-induced maternal effects result in enhanced immune responsiveness across generations.

Rebeca B. Rosengaus^1*^, Nicole Hays^2^, Colette Biro^2^, James Kemos^2^, Muizz Zaman^2^, Joseph Murray^2^, Bruck Gezahegn^2^, Wendy Smith^2^

^1^ Department of Marine & Environmental Sciences, Northeastern University, 134 Mugar Bldg, 360 Huntington Avenue, Boston, MA 02115. 617-3737032.

^2^ Department of Biology, Northeastern University, 134 Mugar Bldg, 360 Huntington Avenue, Boston, MA 02115

*Correspondence author Email: r.rosengaus@neu.edu

**Rationale behind experimental design**

1. ***Choice of pathogen***

*Serratia marcescens* bacterium is likely encountered by developing larvae during herbivory and during the fifth and final larval stage when larvae wander and/or when they pupate subterraneously.

1. ***Choice of pupal developmental stage***

Oocyte maturation begins in lepidopteran females, including *M. sexta*, during pupation ([Nijhout & Riddiford 1974](#_ENREF_11); [Ma *et al.* 1988](#_ENREF_10)) and thus, injections at the pupal developmental stage included the time frame when maternal contributions and the assumed maternal effects would be most prominent. Late-stage pupae were chosen over adults, as pupal injection minimizes handling while still permitting a relatively short lag between injection and oviposition.

1. ***Choice of injections as the delivery mode for Serratia***

Immune-elicitation via injection (rather than consumption of pathogenic bacteria), bypassed the digestive system and reduced possible negative effects of ingestion of *Serratia* on the normal gut microbiome, which has been shown to play a significant role in the overall well-being (including immunocompetence) of a diverse group of organisms ([Thaiss *et al.* 2016](#_ENREF_16)).

***(d) Serratia cultures***

*S. marcescens* was cultured by following previously published protocols ([Hamilton, Lejeune & Rosengaus 2010](#_ENREF_4)). Briefly, the bacteria was cultured overnight in tryptic soy broth to a concentration of 10^8^ cells/mL. Bacterial were pelleted and re-suspended in sterile insect Burns-Tracey solution (BTS, ([Ludwig, Tracey & Burns 1957](#_ENREF_9))). Half of this live bacteria suspension was boiled in a water bath for 20 minutes to create a heat-killed bacterial suspension with a high density of immune-elicitors (peptidoglycans and lipopolysaccharides) corresponding to the density of the original live bacterial stock suspension ([Hamilton, Lejeune & Rosengaus 2010](#_ENREF_4); [Rosengaus, Malak & McKintosh 2013](#_ENREF_12)). The other half of the live *Serratia* stock suspension was further diluted down to a previously established non-lethal dose of 400 bacteria cells/µL.

***e) Somatic and reproductive trade-offs in mothers***

We feel confident that maternal treatment impacted the mother’s pupal physiology. Both cuticular injury and even more influential, the exposure to immune-elicitors or life-*Serratia* set the stage for trade-offs between mothers’ somatic/immune maintenance and their reproductive systems. For example, pupae injected with heat-killed and live *Serratia* tended to have lower median mass (mean ranks = 14.4 and 14.5, respectively) relative to that of naïve and saline-injected pupae (mean ranks = 21.2 and 22.7, respectively; *X^2^* = 5.9, df = 3, *p* = 0.1, Kruskal-Wallis test). Moreover, those saline-, heat-killed and live-*Serratia* treated females that successfully eclosed also tended to have lower fecundity (mean ranks of oviposited embryos = 31.6, 22.5 and 33.5, respectively) than that recorded for naïve mothers (mean rank = 37.3; *X^2^* = 5.8, df = 3, *p* = 0.1, Kruskal-Wallis test). The fact that these measures of maternal biological “wellbeing” were affected by treatment strengthen the validity and reliability of the reported maternal effects on the progeny’s morphometric, developmental and immunological responses.

***(f) Additional statistical analyses on the embryo quality***

In addition to the analysis on embryo quality described in the main article, multiple pairwise analyses comparing differences in embryo mass (Table S1) and embryo volume (Table S2) as a function of maternal treatment [while accounting for the fact that multiple embryos were oviposited by the same mother (random factor)] were generated through linear mixed-effect models using Bonferroni corrections.

**Table S1- Statistical significance of pairwise comparisons in relation to embryo mass after controlling for the fixed effects of embryonic volume and the random effect of maternal identity. * denotes significance at *p* = 0.02, ** *p* = 0.001 and *** at *p* ≤ 0.0001, NS indicates no significance at *p* > 0.02.**

|  |  | **MATERNAL TREATMENT** | | | |
| --- | --- | --- | --- | --- | --- |
|  |  | **Naive** | **Saline-injected** | **Heat-killed *Serratia* injected** | **Live *Serratia-*injected** |
| **MATERNAL TREATMENT** | **Naive** | --- | *** | NS | *** |
|  | **Saline-injected** | *** | --- | ** | * |
|  | **Heat-killed *Serratia* injected** | NS | ** | --- | *** |
|  | **Live *Serratia-*injected** | *** | * | *** | --- |

**Table S2- Statistical significance of pairwise comparisons in relation to embryo volume as a function of maternal treatment and after controlling for the fixed effect of embryonic mass and the random effect of maternal identity. *** denotes significance at *p* ≤ 0.0001, NS indicates no significance at *p* > 0.05.**

|  |  | **MATERNAL TREATMENT** | | | |
| --- | --- | --- | --- | --- | --- |
|  |  | **Naive** | **Saline-injected** | **Heat-killed *Serratia* injected** | **Live *Serratia-*injected** |
| **MATERNAL TREATMENT** | **Naive** | --- | *** | *** | *** |
|  | **Saline-injected** | *** | --- | NS | *** |
|  | **Heat-killed *Serratia* injected** | *** | NS | --- | *** |
|  | **Live *Serratia-*injected** | *** | *** | *** | --- |

The pairwise comparisons of Tables S1 and S2 together with the analyses of Fig 2 (main text) and the linear mixed-effect models (main text) are generally consistent with the idea that 1) cuticular injury (through puncturing) influences measures of progeny quality (by comparing phenotypic traits of offspring from saline injected vs. naïve mothers) and 2) maternal exposure to bacterial pathogens (after controlling for multiple variables that can affect progeny quality) can significantly influence the physical phenotypic traits of embryos above and beyond the effects due to cuticular injury (comparing progeny from heat-killed and live *Serratia* injected mothers to the maternal saline injected treatment).

***(g) Bleeding and plating of hemolymph for the enumeration of bacteria techniques***

To ensure that we collected the maximum number of bacterial cells, each cold-immobilized larva was first surface sterilized by first dipping in 70% ethanol. Then, larvae were washed in sterile water to remove any ethanol residue that could have killed any of the recovered internal *S. marcescens*. Subsequently, larvae were placed ventral side-up on a sterile wax dissecting plate containing a small depression. The larvae were held in place with sterile insect pins (figure 2 Supplementary Material). To ensure the recovery of the maximum number of bacteria circulating in the hemocoel (either suspended in the hemolymph and/or lodged on or between internal organs), an incision was made with sterile dissection scissors along the anterior-posterior axis of the larva. Three additional cuts were made laterally to the initial cut to reveal most of the internal organs. 100 µL of sterile saline were added over the preparation which ultimately pooled in the wax depression. This volume was repeatedly pipetted up and down with the micropipette to create turbulence to dislodge as many adhered bacteria from the tissues as possible. Two 40 µL samples from each larva were collected, plated on tryptic soy agar (TSA) with the use of sterile glass beads, and incubated at 25°C for at least 24 hours (Fig. 3, Supporting information).

***Serratia loads***

Relative to the larvae of saline-injected maternal treatment (which had an average recovery rate of 551 ± 61.8 CFUs across all bleeding time points), larvae from the heat-killed and live-*Serratia* injected mothers supported significantly lower *Serratia* loads (Fig. 4; average across all bleeding time points = 202 ± 35.6 and 166 ± 24.5 CFUs, respectively).

***(g) Maternal translocation of bacteria during oogenesis***

We attempted to identify one of the possible mechanisms by which TGIP occurred, namely the vertical transmission of live bacteria across generations. Such trans-ovarian infection could be responsible for eliciting the embryo’s own immunological responses in a strain-specific manner ([Freitak *et al.* 2014](#_ENREF_2); [Knorr *et al.* 2015](#_ENREF_8)), a required characteristic for TGIP to be of adaptive value ([Sorci & Clobert 1995](#_ENREF_15); [Sadd *et al.* 2005](#_ENREF_13)). One possible vehicle through which translocation of bacteria (or its constituents) could occur is through the incorporation of vitellogenin into the developing embryos. Vitellogenin is not only an important and nutritious lipoprotein precursor of insect egg-yolk that mothers embed into the embryo, but also binds to microbial lipopolysaccharides/peptidoglycans. These microbial-derived fragments (i.e. immune-elicitors) could then trigger the embryo’s own immunological responses ([Salmela, Amdam & Freitak 2015](#_ENREF_14)). Clearly, for this strategy to be successful, insect embryos must mount immune responses, a feat which, in spite their immature state, has been observed in embryos that contain extraembryonic serosal epithelium ([Gorman, Kankanala & Kanost 2004](#_ENREF_3); [Abdel-latief & Hilker 2008](#_ENREF_1); [Jacobs & van der Zee 2013](#_ENREF_6); [Jacobs, Spaink & van der Zee 2014](#_ENREF_5)) (but see ([Jacobs *et al.* 2014](#_ENREF_7))). To this end, the freshly sectioned samples were stained using *S. marcescens* monoclonal antibody [B/N4N from Abcam] diluted at a 1:400, and goat anti-mouse IgG secondary antibody with red fluorophore [Cell Signaling Technology] also diluted to a ratio of 1:400. The slides were visualized under a fluorescent microscope and photographs were taken at 10X magnification. As positive controls, three additional preparations were imaged: a) pure *S. marcescens* CFUs; b) chorion-disrupted embryo contents that were mixed with live *S. marcescens*; and c) first instar larvae immediately after injection with 1.5x10^5^ live *S. marcescens*, as described above. Following imaging, all slides were stored flat in the dark at 4⁰C.

If *Manduca* mothers from the heat-killed and live-*Serratia* treatments allowed the translocation of bacteria from their tissues to their embryos, as has been described in other systems ([Freitak *et al.* 2014](#_ENREF_2); [Knorr *et al.* 2015](#_ENREF_8); [Salmela, Amdam & Freitak 2015](#_ENREF_14)), we would have expected to observe fluorescence in the experimental embryos. This, however, was not the case (Fig. S6, Supporting information) and given that our positive controls fluoresced in the presence of high density of *Serratia*, we conclude that either vertical transmission of this bacterium (or its components) does not take place or that the technique was not sensitive enough to detect low loads of bacteria within the experimental embryos.

**Figure S1: *Manduca sexta* first instar larvae injection**

**
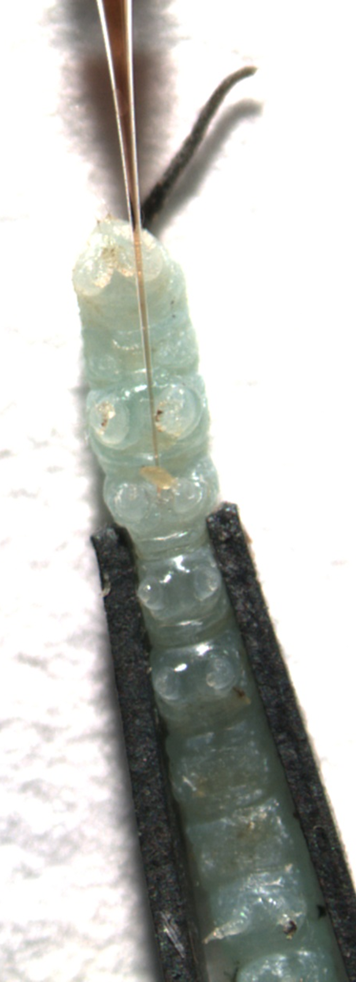
**

**
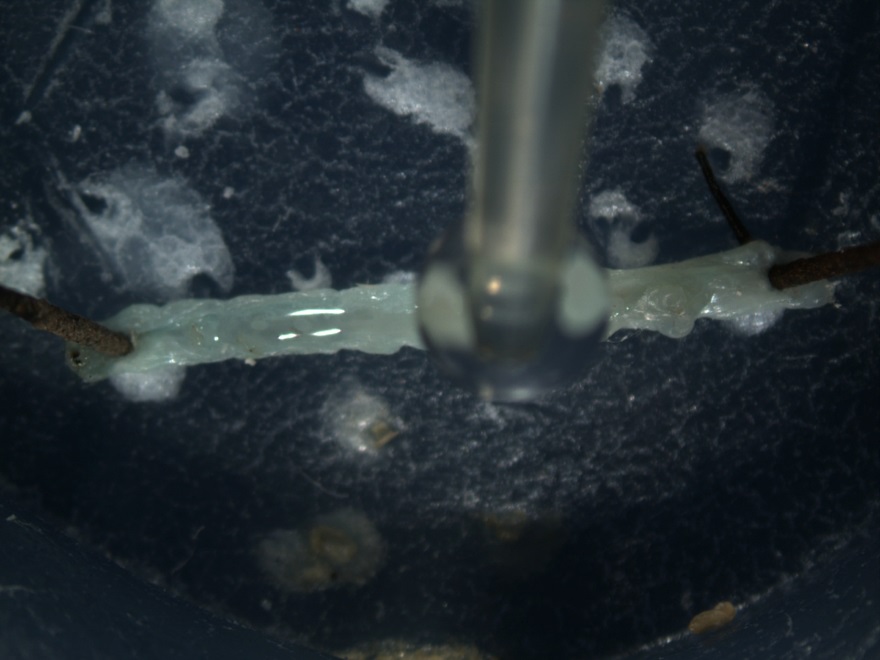

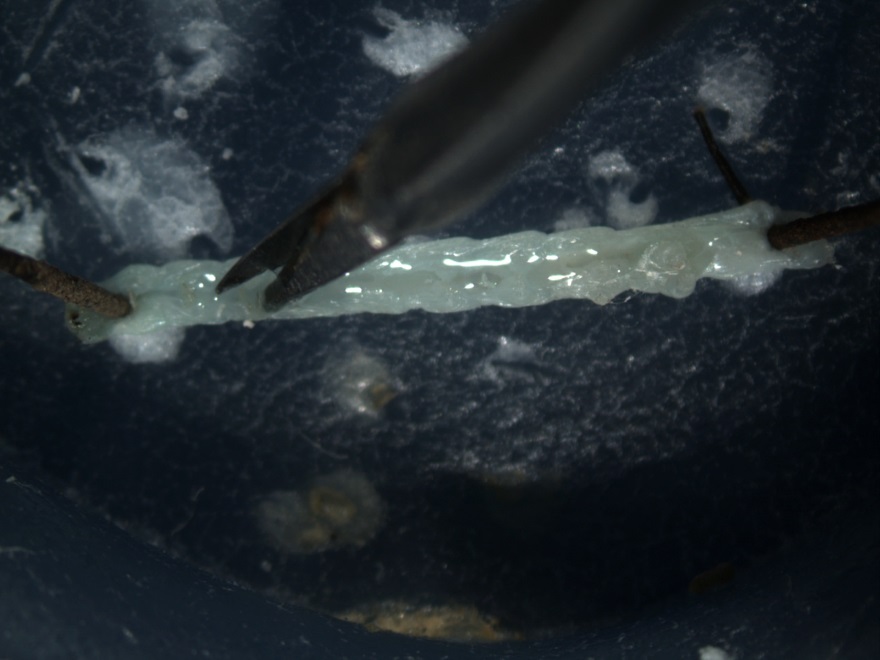
Figure S2. Incision and hemolymph collection of first instar larvae of *Manduca sexta***

**Figure S3. Time course of *Serratia* clearing by first instar larvae injected with live *Serratia*. These specific images represent bacterial loads through time of progeny of saline-injected mothers.**

**
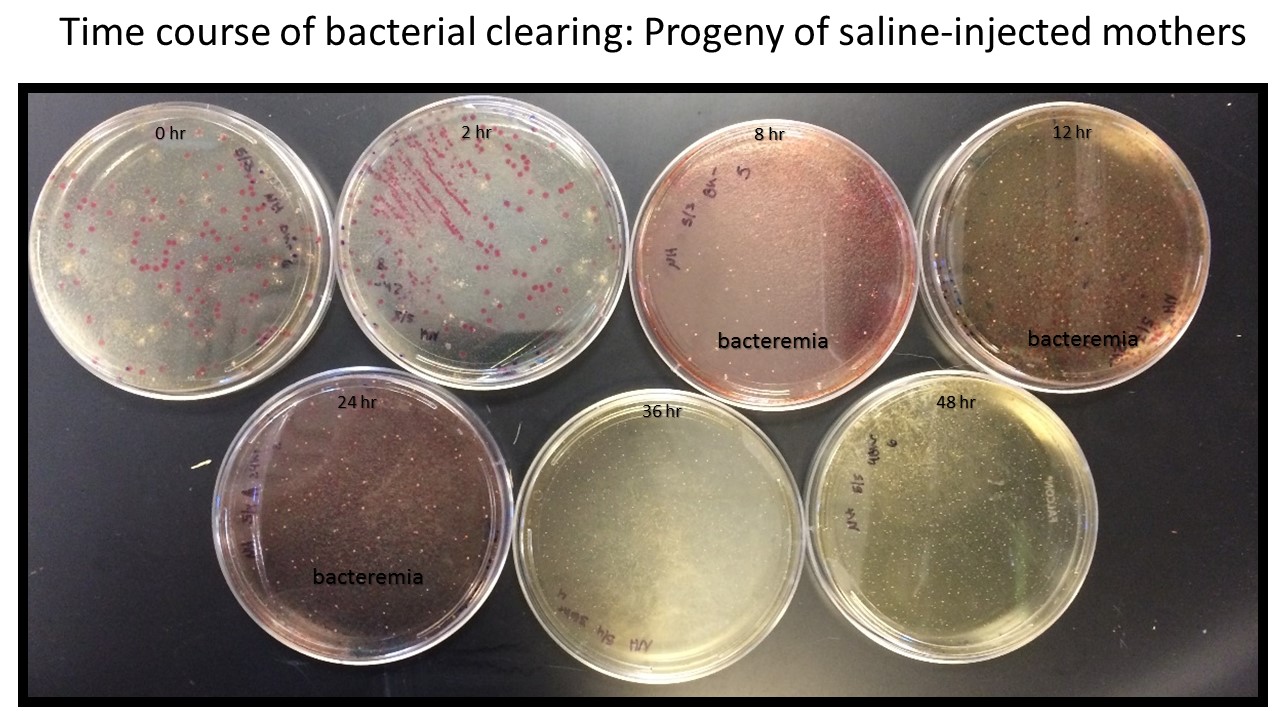
**

**Figure S4. Positive identification of *Serratia* CFUs from plates seeded with hemolymph of challenged larvae through the use of *Serratia* specific primers. None of the white bacteria in the plate showed a positive band on our PCR gel.**

**
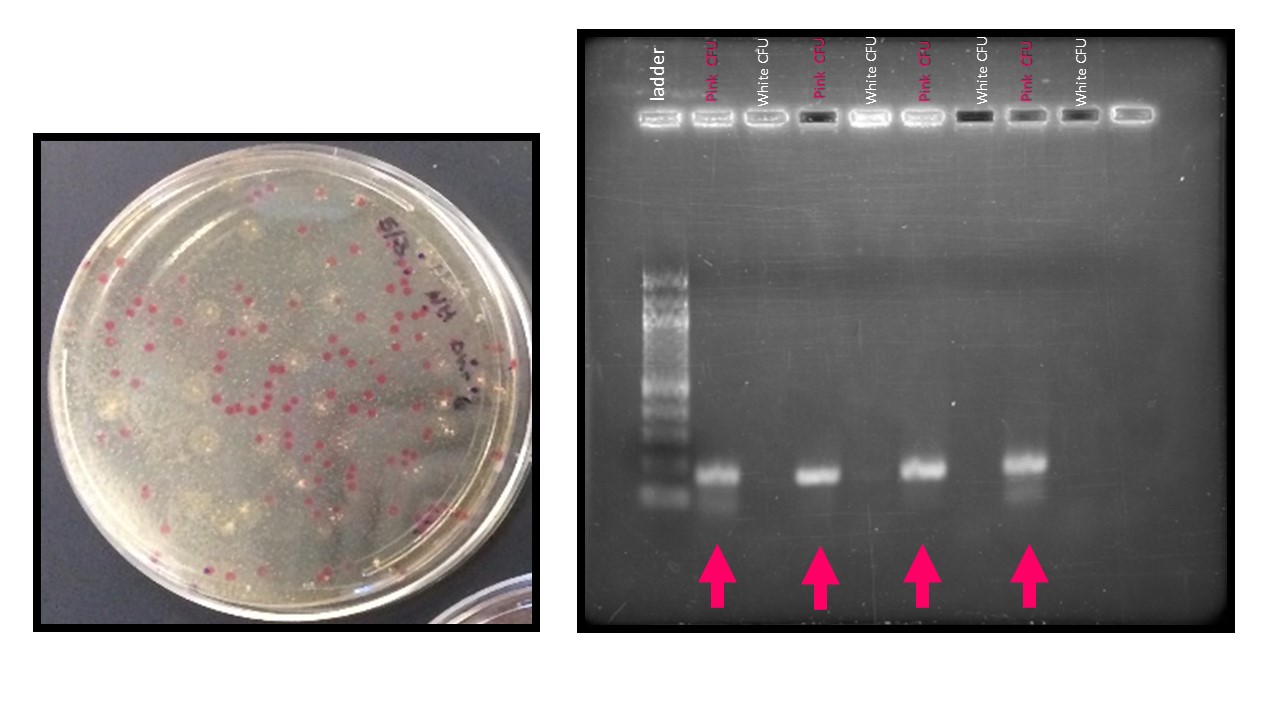
**

**Table S3.** Pairwise comparisons of recovered *Serratia* loads as a function of maternal treatment and incubation time. These comparisons should be seen in conjunction with Fig. 4 main text. * denotes significance at *p* ≤ 0.002 (due to 21 multiple pairwise comparisons) using Mann-Whitney tests after Bonferroni corrections. NS denotes no significant differences (at *p* > 0.002).

|  |  |  |  |  |  |  |  |  |  |
| --- | --- | --- | --- | --- | --- | --- | --- | --- | --- |
|  | **Incubation time** | **Pairwise comparisons** | | | | | | |  |
| **Saline-injected mothers** |  | **0** | **2** | **8** | **12** | **24** | **36** | **48** |  |
|  | **0** | **-** |  |  |  |  |  |  |  |
|  | **2** | **NS** | **-** |  |  |  |  |  |  |
|  | **8** | ***** | ***** | **-** |  |  |  |  |  |
|  | **12** | ***** | ***** | **NS** | **-** |  |  |  |  |
|  | **24** | **NS** | **NS** | ***** | ***** | **-** |  |  |  |
|  | **36** | **NS** | **NS** | ***** | ***** | **NS** | **-** |  |  |
|  | **48** | ***** | **NS** | ***** | ***** | **NS** | ***** | **-** |  |
|  |  |  |  |  |  |  |  |  |  |
|  | **Incubation time** | **Pairwise comparisons** | | | | | | |  |
| **Heat-killed injected mothers** |  | **0** | **2** | **8** | **12** | **24** | **36** | **48** |  |
|  | **0** | **-** |  |  |  |  |  |  |  |
|  | **2** | **NS** | **-** |  |  |  |  |  |  |
|  | **8** | ***** | **NS** | **-** |  |  |  |  |  |
|  | **12** | ***** | **NS** | **NS** | **-** |  |  |  |  |
|  | **24** | **NS** | **NS** | ***** | ***** | **-** |  |  |  |
|  | **36** | **NS** | **NS** | **NS** | **NS** | **NS** | **-** |  |  |
|  | **48** | **NS** | **NS** | **NS** | **NS** | **NS** | **NS** | **-** |  |
|  |  |  |  |  |  |  |  |  |  |
|  | **Incubation time** | **Pairwise comparisons** | | | | | | |  |
| **Live-*Serratia* injected mothers** |  | **0** | **2** | **8** | **12** | **24** | **36** | **48** |  |
|  | **0** | **-** |  |  |  |  |  |  |  |
|  | **2** | **NS** | **-** |  |  |  |  |  |  |
|  | **8** | ***** | **NS** | **-** |  |  |  |  |  |
|  | **12** | **NS** | **NS** | ***** | **-** |  |  |  |  |
|  | **24** | ***** | ***** | ***** | **NS** | **-** |  |  |  |
|  | **36** | **NS** | **NS** | ***** | **NS** | **NS** | **-** |  |  |
|  | **48** | ***** | ***** | ***** | **NS** | **NS** | **NS** | **-** |  |
|  |  |  |  |  |  |  |  |  |  |

**Figure S5. Time course of *Serratia* clearing as a function of time elapsed since challenge and maternal treatment. Although the data are identical to that shown in Figure 2 in the main text the axis here were flipped to better visualize the timing of bacterial clearing. Differences in the number of recovered *Serratia* were analyzed with Kurskal-Wallis tests for each of the bleeding time periods. Circles represent outliers.**

**
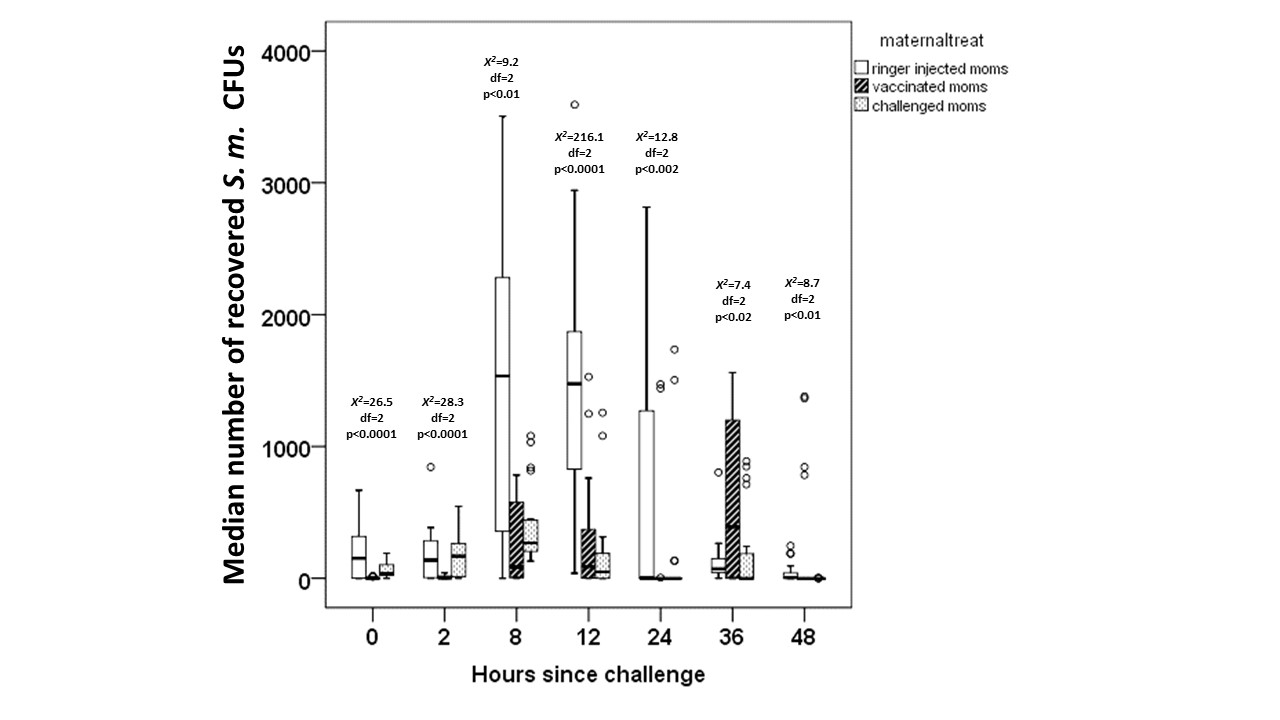
**

**
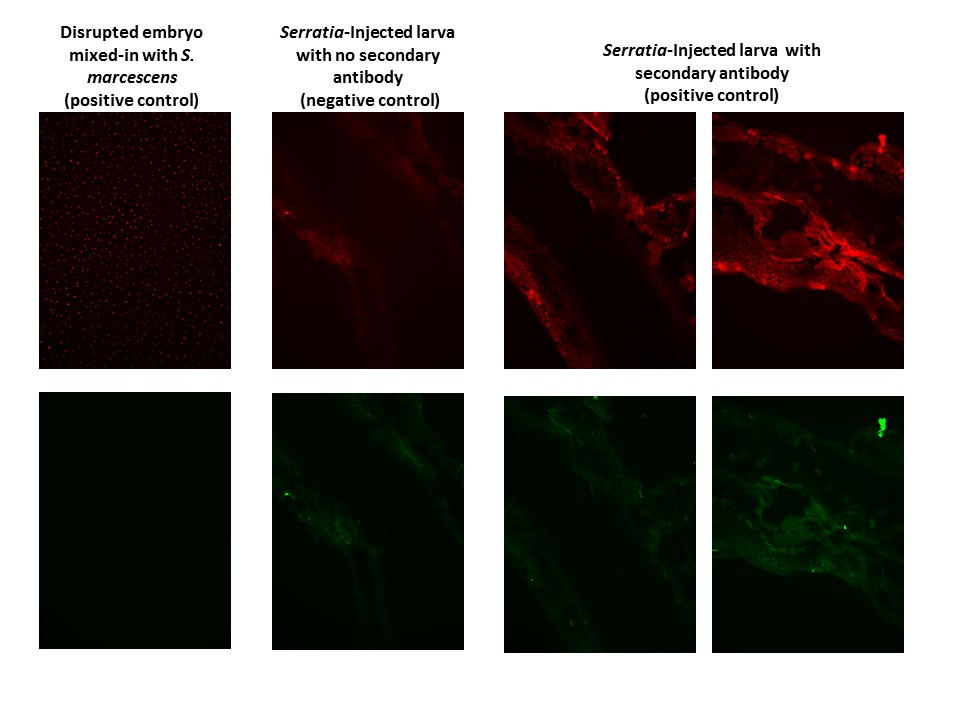
Figure S6. Histo-immunofluorescent samples of positive and negative controls as well as experimental embryos from mothers injected with live *Serratia* bacteria. The samples were viewed under the green laser filter as well to confirm that any red fluorescence observed was not due to auto-fluorescence (green).** **None of the experimental embryos exhibited fluorescence.**

a

a

**
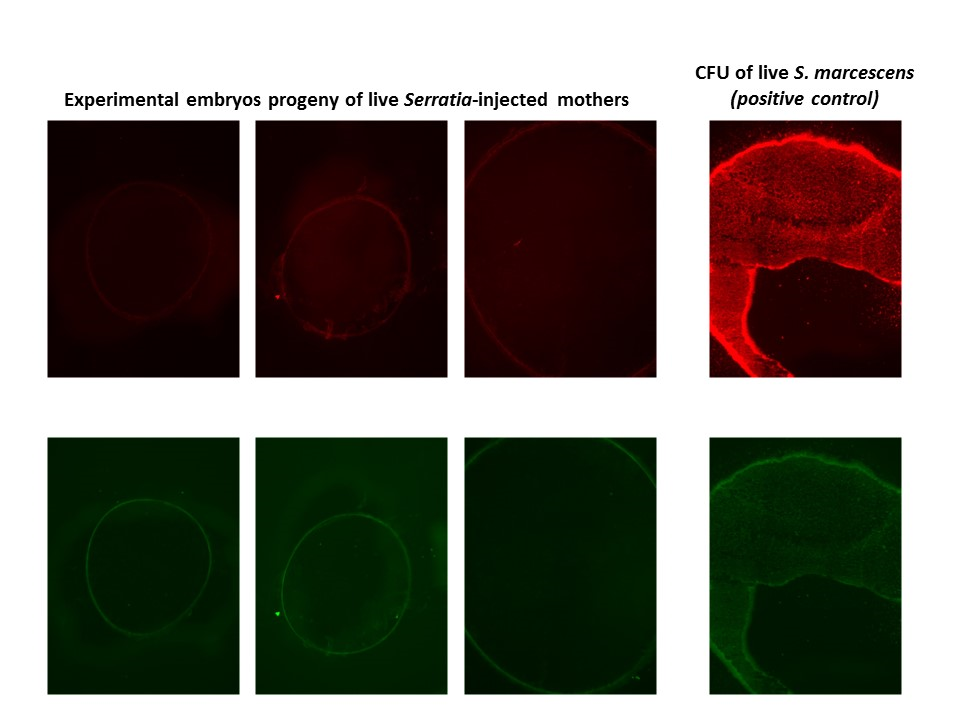
**

**References**

Abdel-latief, M. & Hilker, M. (2008) Innate immunity: eggs of *Manduca sexta* are able to respond to parasitism by *Trichogramma evanescens*. *Insect Biochemistry and Molecular Biology,* **38,** 136-145.

Freitak, D., Schmidtberg, H., Dickel, F., Lochnit, G., Vogel, H. & Vilcinskas, A. (2014) The maternal transfer of bacteria can mediate trans-generational immune priming in insects. *Virulence,* **5,** 547-554.

Gorman, M., Kankanala, P. & Kanost, M. (2004) Bacterial challenge stimulates innate immune responses in extra-embryonic tissues of tobacco hornworm eggs. *Insect Molecular Biology,* **13,** 19–24.

Hamilton, C., Lejeune, B. & Rosengaus, R. (2010) Trophallaxis and prophylaxis: social immunity in the carpenter ant *Camponotus pennsylvanicus. Biology Letters,* **7,** 89-92.

Jacobs, C., Spaink, H. & van der Zee, M. (2014) The extraembryonic serosa is a frontier epithelium providing the insect egg with a full-range innate immune response. *eLife Science,* **3:e04111**.

Jacobs, C. & van der Zee, M. (2013) Immune competence in insect eggs depends on the extraembryonic serosa. *Developmental and Comparative Immunology,* **41,** 263–269.

Jacobs, C., Wang, Y., Vogel, H., Vilcinskas, A., van der Zee, M. & Rozen, D. (2014) Egg survival is reduced by grave-soil microbes in the carrion beetle, *Nicrophorus vespilloides*. *BMC Evolutionary Biology,* **14,** 208.

Knorr, E., Schmidtberg, H., Arslan, D., Bingsohn, L. & Vilcinskas, A. (2015) Translocation of bacteria from the gut to the eggs triggers maternal transgenerational immune priming in *Tribolium castaneum*. *Biology Letters,* **11,** 20150885.

Ludwig, D., Tracey, K. & Burns, M. (1957) Ratios of ions required to maintain the heart beat of the American cockroach, *Periplaneta americana* Linnaeus. *Annals of the Entomological Society of America,* **50,** 244–246.

Ma, M., Sieber, K.-P., Ballarino, J. & Wu, S.-J. (1988) ELISA and Monoclonal Antibodies. . *Immunological Techniques in Insect Biology* (eds G. LI & M. TA), pp. 43-73. Springer-Verlag, Nwe York.

Nijhout, M.M. & Riddiford, L.M. (1974) The control of egg maturation by juvenile hormone in the tobacco hornworm moth, *Manduca sexta*. *Biol Bull,* **146** 377-392.

Rosengaus, R.B., Malak, T. & McKintosh, C.H. (2013) Immune-Priming in Ant Larvae: Social Immunity Does Not Undermine Individual Immunity. *Biology Letters,* **9,** 20130563.

Sadd, B.M., Kleinlogel, Y., Schmid-Hempel, R. & Schmid-Hempel, P. (2005) Trans-generational immune priming in a social insect. *Biology Letters,* **1,** 386-388.

Salmela, H., Amdam, G.V. & Freitak, D. (2015) Transfer of immunity from mother to offspring is mediated via egg-yolk protein vitellogenin. *PLoS One,* **11,** e1005015.

Sorci, G. & Clobert, J. (1995) Effects of maternal parasite load on offspring life‐history traits in the common lizard *(Lacerta vivipara*). *Journal of Evolutionary Biology,* **8,** 711-723.

Thaiss, C.A., Zmora, N., Levy, M. & Elinav, E. (2016) The microbiome and innate immunity *Nature Reviews Microbiology,* **535,** 65–74.
